# Higher cyclosporine-A concentration increases the risk of relapse in AML following allogeneic stem cell transplantation from unrelated donors using anti-thymocyte globulin

**DOI:** 10.1038/s41598-023-50105-4

**Published:** 2023-12-20

**Authors:** Mikael Lisak, Malin Nicklasson, Robert Palmason, Stina Wichert, Cecila Isaksson, Per-Ola Andersson, Jan-Erik Johansson, Stig Lenhoff, Mats Brune, Markus Hansson

**Affiliations:** 1 Department of Hematology and Coagulation, Sahlgrenska University Hospital, Bruna stråket 5, plan 5, 413 45 Gothenburg, Sweden; 2 Sahlgrenska Academy, Gothenburg University, Gothenburg, Sweden; 3 Department of Hematology, Skane University Hospital, Lund, Sweden; 4 Department of Hematology, Norrland University Hospital, Umeå, Sweden

**Keywords:** Acute myeloid leukaemia, Bone marrow transplantation

## Abstract

Cyclosporine-A (CsA) is used to prevent acute graft-versus-host disease (aGvHD). European Society for Blood and Marrow transplantation (EBMT) recommends a CsA target serum concentration of 200–300 µg/L during the first month after allogeneic hematopoietic stem cell transplantation (HSCT). With this study, we investigated whether a median CsA concentration > 200 µg/L (CsA_high_) the first month after HSCT, compared to ≤ 200 µg/L (CsA_low_), increased the relapse risk of acute myloid leukemia (AML), using unrelated donors (URD) and antithymocyte globulin (ATG). Data was collected from 157 patients with AML, transplanted 2010–2016. The cumulative incidence of relapse (CIR) at 60 months was 50% in the CsA_high_ versus 32% in the CsA_low_ group (p = 0.016). In univariate analysis, CsA_high_ versus CsA_low_ (p = 0.028), 10-unit increase of CsA as a continuous variable (p = 0.017) and high risk disease (p = 0.003) were associated with higher CIR. The results remained after adjusting for disease risk. Death following relapse occurred more frequently in the CsA_high_ group (p = 0.0076). There were no significant differences in rates of aGvHD, chronic GvHD (cGvHD), EBV/CMV-infections or overall survival (OS) between the two groups. In conclusion, we found that a median CsA concentration > 200 µg/L, the first month after HSCT, results in higher CIR of AML when combined with ATG.

## Introduction

Allogeneic hematopoietic stem cell transplantation (HSCT) is an effective treatment to prevent relapse for patients with acute myloid leukemia (AML). However, relapse remains the leading cause of death after HSCT^1–3^. Graft-versus-host disease (GvHD) is a common complication after HSCT despite the use of prophylactic measures, and can potentially lead to additional morbidity^4–7^, increased non-relapse mortality (NRM) and reduced overall survival (OS)^8,9^.

Cyclosporine-A (CsA) is commonly used for the prevention of acute GvHD (aGvHD)^10–14^. Higher CsA concentrations, especially during the first month after transplant, including the period of engraftment, have been found to reduce the occurrence and severity of aGvHD^10,14–17^. The European Society for Blood and Marrow transplantation (EBMT) recommends targeting of CsA serum concentration at 200–300 µg/L during the first month after HSCT as prophylaxis against aGvHD^18^. However, some studies have found a correlation between higher CsA concentration and relapse of hematological malignancies^19–22^. Antithymocyte globulin (ATG) is an immunosuppressive agent that prevents or alleviates GvHD, particulary reducing the incidence and severity of chronic GvHD (cGvHD)^23–25^. Historically, ATG has mostly been used when involving unrelated donors (URD), but in recent years also with allografts from related donors (RD)^24,26^. While ATG is frequently used as prophylaxis against cGvHD^27^, there are conflicting evidence regarding its effect on relapse incidence, and there is no clear consensus on optimal dosage in different transplantation settings^25,28–30^. Higher ATG dosages have been linked to increased relapse risk^31^, and therefore, there is concern that the T-cell depletion may impair the Graft-versus-Leukemia effect (GvL).

Nevertheless, CsA, often in combination with methotrexate (MTX) and ATG, is still considered a cornerstone drug for the prevention of aGvHD and graft rejection^18^.

To our knowledge, no study has specifically analyzed the impact of CsA exposure on the risk of AML relapse when combined with ATG. Therefore, the aim of this study was to investigate whether a higher level of CsA blood concentration during the first month after HSCT (> 200 µg/L; CsA_high_ versus ≤ 200 µg/L; CsA_low_), in combination with ATG, is associated with increased incidence of AML relapse.

## Methods

### Patients

This retrospective study recruited adult patients with AML allografted between 2010 and 2016 at three Swedish transplant centers (Sahlgrenska University Hospital, Gothenburg, Skåne University Hospital, Lund and Norrland University Hospital, Umeå).

The inclusion criterias were: (1) AML diagnosis, (2) age ≥ 18 years, (3) HSCT with an URD between 2010 and 2016, (4) stem cell source being bone marrow or peripheral blood stem cells (PBSC), (5) reduced or myeloablative conditioning (RIC, MAC) and (6) ATG, MTX and CsA as GvHD prophylaxis.

The exclusion criterias were: (1) haploidentical donor, (2) cord blood cell transplant, (3) conditioning with total lymphoid irradiation, (4) pre-transplant alemtuzumab in conditioning or less than two months before HSCT, (5) mycophenolate (within 30 days post-HSCT) and (6) CsA treatment < 30 days.

The study was approved by the Ethic Review Board of Gothenburg (“Regionala etikprövningsnämnden i Göteborg”) (Dnr 144-18), and for this retrospective study, informed consent was waived by “Regionala etikprövningsnämnden i Göteborg”. All research was performed in accordance with relevant guidelines/regulations and in accordance with the Declaration of Helsinki.

### Data collection

Clinical data were collected from medical records.

### Immunosuppressive treatment

All patients received ATG (Thymoglobuline^®^ or ATG-Fresenius^®^/Grafalon^®^). In addition, a short course of intravenous MTX(2–4 daily doses, 16–45 mg/m^2^, between day + 1 to + 11 post HST) was administrated as GvHD prevention to all patients (Table 1).

**Table 1:** Background characteristics (n = 157).

Characteristic	CsA conc ≤ 200 µg/L (n = 87)	CsA conc > 200 µg/L (n = 70)	P value
Age at alloSCT, median (range), years	56 (19–71)	51.5 (18–71)	0.26
Female gender, n (%)	38 (44)	32 (46)	0.80
HCT-CI score, n (%)^§^			
0–2	63 (72)	47 (68)	0.52
3–5	23 (26)	20 (29)	
≥ 6	1 (1)	2 (3)	
Disease risk group, n (%)^#^			
Intermediate	26 (30)	21 (30)	0.99
High	61 (70)	49 (70)	
Disease stage at HSCT, n (%)^#^			
Non-CR	10 (11)	4 (6)	0.27
Minimal residual disease at HSCT, n (%)^§^			
Negative	38 (44)	27 (39)	0.93
Positive	8 (9)	6 (9)	
Not done	40 (46)	37 (53)	0.52
Stem cell source, n (%)			
BM	8 (9)	4 (6)	0.67
PBSC	79 (91)	66 (94)	
HLA matching, n (%)^§^			
10/10	73 (84)	54 (77)	0.22
≤ 9/10	13 (15)	16 (23)	
≤ 7/8	8 (9)	7 (10)	0.88
Gender matching (patient/donor), n (%)			
Male/female	4 (5)	7 (10)	0.22
All other combinations	83 (95)	63 (90)	
CMV-IgG (patient/donor), n (%)^§^			
Positive/negative	29 (34)	17 (25)	0.22
Positive/positive	36 (42)	30 (43)	0.84
Negative/positive	2 (2)	3 (4)	0.66
Negative/negative	19 (22)	19 (28)	0.43
Donor age, median (range), years	25 (18–54)*	27 (18–59)	0.24
Creatinine clearence, n (%)			
eGFR^~^, median (range) (ml/min) day − 1 from HSCT	92 (56–154)	91.5 (64–129)	0.84
eGFR^~^, median (range) (ml/min) day + 21 from HSCT	74 (26–132)	78.5 (35–126)	0.26
Conditioning, n (%)			
RIC Flu150-180 + Bu8/BuS 6.4 (n = 79) Flu150 + Treo42 (n = 5)	48 (55)	31 (44)	0.18
MAC Cy120 + TBI 10-12 Gy (n = 9) Cy100-120 + Bu16/BuS9.6–12.8 (n = 34) Flu150-180 + Bu16/Bus9.6–12.8 (n = 30)	39 (45)	39 (56)	
ATG, n (% of ATG)			
ATG-Fresenius™/Grafalon™	17 (20)	13 (19)	0.88
30 mg/kg	2 (12)	1 (8)	1.0
40 mg/kg	15 (88)	12 (92)	
Thymoglobulin™	70 (80)	57 (81)	0.88
4–5 mg/kg	53 (76)	37 (65)	0.18
6–8 mg/kg	17 (24)	20 (35)	
MTX, n (% of MTX)			
MTX median total dose (range) (mg/m^2^)	35 (16–45)	35 (16–45)	0.003
Median CsA concentration			
CsA conc, median (range) (µg/L)	180 (140.5–200)	215 (200.5–260)	< 0.001

~ eGFR measured with the Lund–Malmö revised (LMR) equation^53^.

*CsA* cyclosporine-A, *HCT-CI* hematopoietic cell transplantation comorbidity index, *CR* complete remission, *BM* bone marrow, *PBSC* peripheral blood stem cells, *CMV* cytomegalovirus, *eGFR* estimatied glomerular filtratrion rate, *RIC* reduced intensity conditioning, *MAC* myeloablative conditioning, *Flu* fludarabine, *Bu* busulfan (orally), *BuS* busulfan (intravenously), *Cy* cyclophosphamide, *Treo* treosulfan, *TBI* total body irradiation, anti-thymocyte globulinacute, *MTX* methotrexate.

^§^Data missing for one patient.

*Data missing for two patients.

^#^Risk categorization according to Supplementary 1.

### Cyclosporine

Initially, the patients routinely received CsA intravenously and later switched to oral formulation when tolerated. CsA was administered twice daily and dosage was adjusted to intended concentrations (usually between 150 and 250 µg/L). During the hospitalization period, the trough whole blood CsA samples were collected daily, 12 h after the prior dose and immediately before the morning dose, both after intravenous and oral administration. See Supplementary 1 regarding methods to analyze CsA concentration. Markedly divergent concentrations were excluded. All CsA concentrations the first 30 days after HSCT were registered from which the median CsA concentration was calculated for each patient.

### AML-relapse risk categorization

The risk of relapse at transplantion was categorized into low, intermediate or high risk according to a risk categorization manual (Supplementary 2) based on the Swedish National Guidelines for AML^32^. The risk was determined by using cytogenetics and mutational status, when available, as well as disease-related factors and treatment response.

Minimal residual disease (MRD) status was not included in the risk categorization due to lack of information about MRD in many patients at the time of this study. Though, for the patients with available pre-transplant MRD status, its influence on relapse risk was analyzed separately. In these cases, immunophenotyping was almost exclusively (92%) used to assess MRD, with the cut-off ≥ 0,1% regarded as positive. In a few cases PCR (NPM1 mutation) was used to assess MRD.

### Graft-versus-host disease

Acute GvHD was based on medical records and defined and graded according to the modified Glucksberg criteria^33^. The National Institute of Health (NIH) Consensus Development Project 2014 guidelines were used to define and score cGvHD in each involved organ^34^.

Graft-versus-host disease was registered according to the criteria, within the specified time period or until relapse, re-transplation or death.

### Conditioning intensity

Reduced intensity and myeloablative condtitioning w defined according to Bacigalupo et al.^35^. Total Body Irradiation ≥ 8 Gy fractionated and Busulfan > 8 mg/kg orally (Bu) or > 6,4 mg/kg intravenous (BuS) was regarded as MAC. Treosulfan-based conditioning was defined as RIC (non-myeloablative), when the total treosulfan dose was 30 g/m^2^, and as MAC (toxicity-reduced) when the total dose was 42 g/m^2^.

### Study endpoints

The primary endpoint was the cumulative incidence of relapse (CIR) at 60 months after HSCT. Secondary endpoints were aGvHD (any grade, grade 2–4 and 3–4), cGvHD (any grade within 12 months and moderate/severe cGvHD within 24 months post-HSCT), NRM, relapse-free survival (RFS), time to relapse (TTR) and OS.

Non-relapse mortality was defined as death without previous occurrence of relapse. RFS was defined as survival without occurrence of relapse or death of any cause. Time to relapse was defined as time to first evidence of relapse.

### Statistical analysis

The median CsA concentration was used for analyses due to skew distribution of values. The Cyclosporine-A exposure was dichotomized by cut-off based on EBMT recommendation^18^; CsA_high_ > 200 µg/L and CsA_low_ ≤ 200 µg/L. The Cyclosporine-A exposure was also analyzed by using concentration as a continuous variable and by sectioning the patients into quartiles (based on lowest to highest concentration). Baseline characteristics in the CsA_high_ and CsA_low_ groups were compared using Chi-square (or Fisher´s Exact) test for categorical variables, Mann–Whitney U-test for ordinal data and if skewed distribution, or the Student’s t-test for continuous and normally distributed variables.

The median follow-up time was estimated by the reverse Kaplan–Meier method^36,37^.

Competing event analysis was used to assess CIR and NRM^38^. The Fine-Gray subdistribution hazard model was used to estimate the incidence of outcomes over time in the presence of competing risks. Gray’s test for subdistribution hazards has been used for comparing cumulative incidence functions^39,40^. Death was labelled as competing event in the CIR analysis and in RFS, while death was censored in TTR. In the NRM analysis, relapse was the competing event. Logistic regression was used to compare effect of different quartiles of CsA concentration on secondary endpoints. Overall survival was analyzed with Kaplan–Meier and log-rank testing for group comparisons. Uni- and multivariate analyses were made with Cox regression. To analyze if there was an interaction effect between two predicting variables, a likelihood-ratio test was used comparing regression models with and without the interaction term. The non-linear effect of CsA concentration on relapse was modelled by a spline function in a flexible parametric survival model (Supplementary Fig. 1). Reference point 1.0 of CsA concentration for the hazard ratio was chosen to 140 µg/L. The stpm2 macro developed by Royston and Lambert was used for the analyses^41^. P-values less than 0.05 were considered significant. For most analyses concerning comparison of background characteristics, the SPSS version 24 (IBM^®^ SPSS^®^ Statistics, NY, USA) was used. The Kaplan–Meier calculations and cumulative incidence analyses were performed with Stata for Mac, version 17.0 (StataCorp^®^, TX, USA).

## Results

After an initial screening of 233 patients with AML allografted between 2010 and 2016 at three Swedish transplantation centra, 157 fulfilled the inclusion criteria. Median age at HSCT was 54 years (range: 18–71) and 45% were females. Peripheral blood stem cells was the most common stem cell source (92%). Baseline characteristics were similar between the CsA_high_ and CsA_low_ group, see Table 1. Despite that the methotrexate median total dose and range were the same in the CsA_high_ and CsA_low_ group, the distribution was skew in the former group, resulting in a significant difference between the groups, see Table 1.

The median CsA concentration day 0–30 after HSCT amongst all patients was 198 (range: 140.5–260) µg/L, and 180 (range: 140.5–200) versus 215 (range: 200.5–260) µg/L, in the CsA_low_ and the CsA_high_ group respectively. The median follow-up time for all patients was 57.5 months (95% confidence interval [CI], 52.9–64.6).

### Relapse

**Figure 1:**
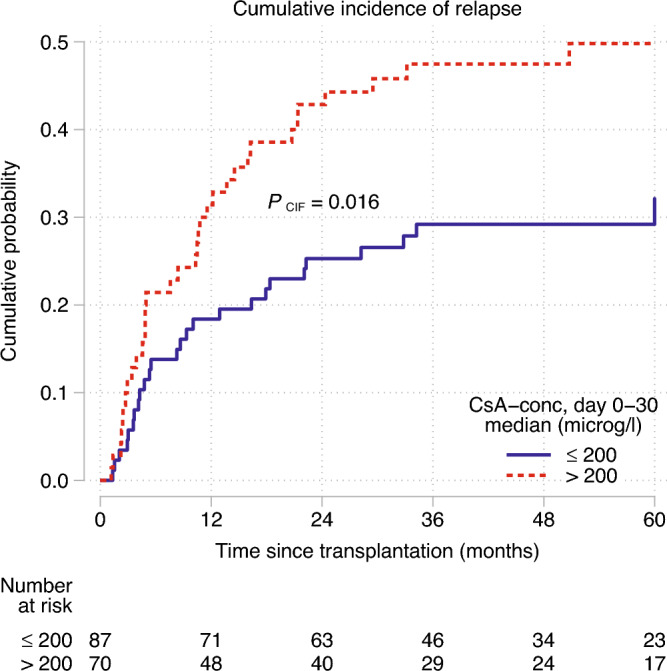
The cumulative incidence of relapse (CIR) during the first 60 months post-HSCT, compared between CsA_high_ and CsA_low_. Competing event is death.

Sixty-two patients (39%) relapsed during the follow up period, 28 (32%), in the CsA_low_ group and 34 (49%) in the CsA_high_ group (p = 0.037) (Table 2). The 60-month CIR was 50% (95% CI, 38– 62) in the CsA_high_ group compared to 32% (95% CI, 23–44) in the CsA_low_ group (p = 0.016), see Fig. 1, and 40% (95% CI, 32–48) in the whole cohort.

**Table 2:** Results (n = 157).

Variable	CsA conc ≤ 200 µg/L (n = 87)	CsA conc > 200 µg/L (n = 70)	P value
Acute GvHD, n (%)			
Any grade	53 (61)	37 (53)	0.31
Grade 1	26 (30)	21 (30)	0.69
Grade 2	20 (23)	14 (20)	
Grade 3	5 (6)	2 (3)	
Grade 4	2 (2)	0 (0)	
Grade 0–1	60 (69)	54 (77)	*
Grade 2–4	27 (31)	16 (23)	
Grade 0–2	80 (92)	68 (97)	*
Grade 3–4	7 (8)	2 (3)	
Chronic GvHD within 12 mos after HSCT, n (%)			
Any grade	51 (59)	45 (64)	*
Chronic GvHD within 24 mos after HSCT, n (%)			
Moderate–severe	19 (22)	20 (29)	*
CMV treatment within first yr after HSCT, n (%)			
Yes	31 (36)	27 (39)	0.83
EBV treatment within first yr after HSCT, n (%)			
Yes	15 (17)	15 (21)	0.51
Relapse, n (%)	28 (32)	34 (49)	*
Death, n (%)	38 (44)	33 (47)	*
Causes of death, n (% of deaths)			
Relapse	22 (58)	29 (88)	0.0076
GvHD or related infections	4 (11)	2 (6)	0.69
Infection	5 (13)	2 (6)	0.44
Other	7 (18)	0 (0)	0.013

*GvHD* graft-versus-host disease, *HSCT* allogeneic stem cell transplantation, *CMV* cytomegalovirus, *EBV* Epstein–Barr-virus, *CI* confidence interval.

*See in the chapter “Results”.

Univariable analysis with Cox regression confirmed a higher incidence of relapse in the CsA_high_ versus CsA_low_ group [hazard ratio (HR), 1.77; 95% CI, 1.06–2.95 p = 0.028]. Additionally, when using CsA concentration as a continuous variable, every 10-unit increase of CsA concentration increased the risk of relapse (HR, 1.15; 95% CI, 1.02–1.28; p = 0.017).

Besides CsA concentration, high-risk disease was the only factor associated with increased 60-month CIR (HR, 2.84; 95% CI, 1.44–5.61; p = 0.003). The median CsA concentrations did not differ between the risk groups (intermediate risk:198.0 µg/L, high risk:198.3 µg/L). When adjusting for disease risk in multivariable analysis, relapse incidence remained higher in the CsA_high_ versus CsA_low_ group [hazard ratio (HR), 1.78; 95% CI, 1.07–2.96 p = 0.028] and so did every 10 unit-increase of CsA concentration (HR, 1.14; 95% CI, 1.02–1.20; p = 0.017).

A likelihood-ratio test showed no significant interaction between the median CsA concentration and disease risk (p = 0.25). Furthermore, MRD status was not significantly associated with relapse risk.

The median CsA concentration was lower amongst patients without relapse compared to those with relapse; 194 (range 140.5–235) µg/L versus 202.5 (range 144.5–260) µg/L (p = 0.019). The quartile of patients with the highest CsA concentrations (210–260 µg/L), had an increased rate of relapse (HR, 2.05; 95% CI, 1.01–4.13; p = 0.046) compared to the quartile with lowest concentrations (140.7–177.5 µg/L).

To analyze the chosen CsA concentration cut-off at 200 µg/L, a non-linear risk analysis was made confirming the cut-off being appropriate (Supplementary Fig. 1). Besides, the CsA exposure with mean concentration for each patient was analyzed (data not shown). In general, the results pointed in the same directions.

### Acute and chronic GvHD

No significant difference was seen between the CsA_high_ versus CsA_low_ group in the rate (p = 0.31) and severity of aGvHD; grade 2–4 [odds ratio (OR), 0.66; 95% CI, 0.32–1.35; p = 0.26] and grade 3–4 (OR, 0.34; 95% CI, 0.07–1.67; p = 0.18). Additionally, there was no difference in aGvHD grade 2–4 when the quartile of patients with highest CsA concentrations was compared to the quartile with lowest concentrations (HR, 0.44; 95% CI, 0.17–1.15; p = 0.093). Neither did CsA_high_ and CsA_low_ differ in the rate of cGvHD; any grade within 12 months post-HSCT (OR, 1.27; 95% CI, 0.66–2.43; p = 0.47) or moderate/severe cGvHD within 24 months post-HSCT (OR, 1.43; 95% CI, 0.69–2.96; p = 0.33).

### Reactivation of Epstein–Barr virus (EBV) and cytomegalovirus (CMV)

No differences were seen in the in incidence of clinical significant reactivations of EBV or CMV.

### NRM, RFS, TTR and overall survival

**Figure 2:**
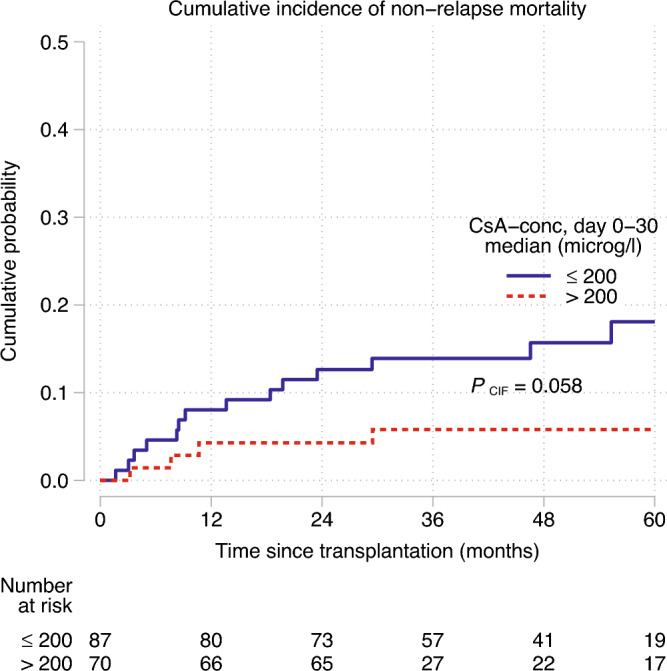
The non-relapse mortality during the first 60 months post-HSCT, compared between CsA_high_ and CsA_low_. Competing event is relapse.

The cumulative incidence of NRM was 12.5% at 60-months in the whole cohort, and 18.1% (95% CI, 10.9–29.3) in the CsA_low_ group compared to 5.8% (95% CI, 2.2–14.7) in the CsA_high_ group (p = 0.058), see Fig. 2.

**Figure 3:**
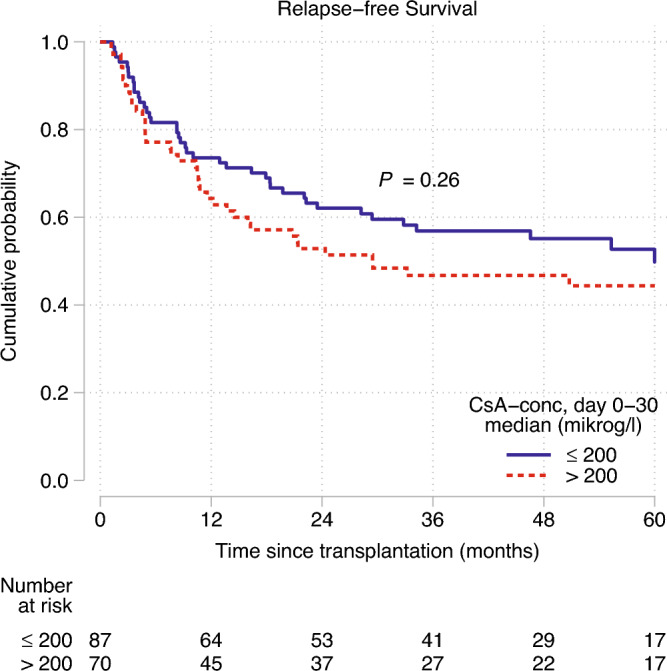
The relapse-free survival during the first 60 months post-HSCT, compared between CsA_high_ and CsA_low_.

The RFS at 60 months was 49.8% (95% CI, 37.3–61.1) versus 44.4% (95% CI, 32.2–55.9) in the CsA_low_ and CsA_high_ group, respectively (p = 0.26), see Fig. 3.

**Figure 4:**
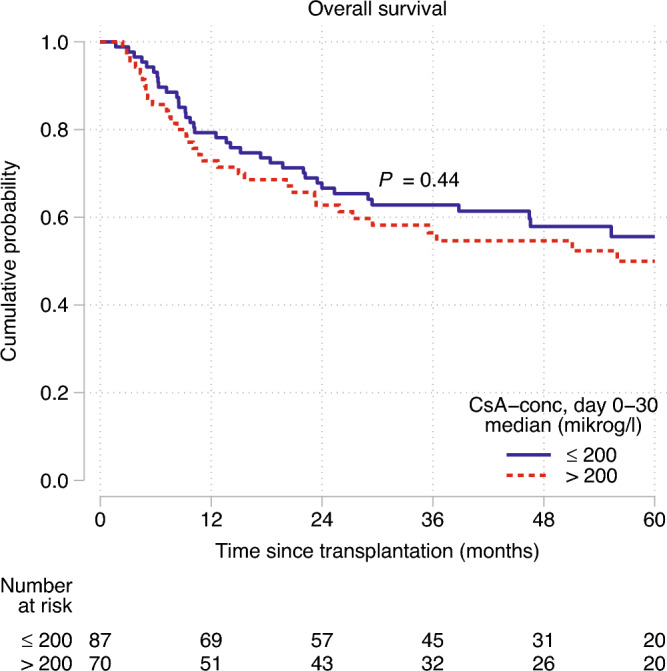
The overall survival during the first 60 months post-HSCT, compared between CsA_high_ and CsA_low_.

Seventy-one patients (45%) died during follow up, without differences in death rates; 44% in the CsA_low_ and 47% in the CsA_high_ group. The 60-month OS for CsA_high_ was 55.6% (95% CI, 43.5–66.0) compared to 50.0% (95% CI, 36.9–61.7) in the CsA_low_ group (p = 0.44), see Fig. 4. The OS for the whole cohort at 24 and 60 months was 65% (95% CI, 57–72) and 53% (95% CI, 44–61), respectively. Relapse was the most common cause of death, 51 of 71 deaths (72%), and was more frequent in the CsA_high_ compared to the CsA_low_ group; 88% versus 58% (p = 0.0076). In the CsA_low_ group, seven patients died from other causes (glioblastoma n = 1, neuroendocrine tumor n = 1, intracranial hemorrhage n = 1, cardiovascular disease n = 1, idiopatic pneumonia syndrome n = 1, unknown n = 2).

Due to excess of deaths of other causes in the CsA_low_ group, a TTR analysis was made where patients with relapse-free deaths were censored as opposed to the calculations of RFS. The 60-month CIR (K-M; relapse-free deaths censored) for CsA_high_ was 51.6% (95% CI, 39.9–64.5) compared to 35.4% (95% CI, 24.7–49.0) in the CsA_low_ group (p = 0.026), see Supplementary Fig. 2.

## Discussion

In this study, we found that median CsA concentration above 200 µg/L, the first month after HSCT, resulted in higher incidence of AML relapse compared to lower concentrations when combined with ATG treatment. No significant differences between the compared groups were found in clinically relevant EBV/CMV reactivations, acute or chronic GvHD, NRM, RFS or OS.

Acute myeloid leukemia is the most common indication for HSCT^42^, with the goal of preventing relapse and increase survival. Nevertheless, relapse, which often occurs within the first year after HSCT^43,44^, is the leading cause of death^1–3^. Factors that determine the risk of relapse include the disease characteristics at diagnosis, treatment response and post-transplant immunosuppressive treatment after HSCT^45–48^. There is no consensus on whether CsA exposure interacts with ATG on the GvL-effect, and consequently the risk of relapse.

In the EBMT recommendation, no clear distinction in targeted CsA concentration is made concerning the use of parallell immunosuppressive drugs, e.g. ATG^18^, potentially hampering the GvL-effect.

A few prior studies have shown a correlation between high early CsA concentration and increased relapse incidence^19,20,22,49^. To our knowledge, this is one of few studies of a uniform cohort, solely including AML-patients allografted with URD and T-cell depleted with ATG, analyzing the impact of early CsA exposure on relapse incidence.

In the study by Craddock et al.^22^, AML patients, only with RIC, were included and alemtuzumab was used as T-cell-depletion. A subanalysis showed that increased median CsA exposure in the first 21 days post-HSCT was associated with an increased risk of relapse and decreased OS. No association between the incidence of aGvHD and CsA concentration was found. These results, in a similar cohort of AML patients, also using T-cell depletion in the conditioning, are in line with the findings in our study.

Additionally, two randomized studies of patients with acute leukemia, one in children^20^ and the other in adults^19^, without T-cell depletion, showed a correlation between higher CsA doses and increased relapse incidence. For the adult patients, a follow-up almost three decades later^21^, showed that the intended GvHD-protection was still offset by increased leukemia relapse, organ toxicity and shorter disease free survival. A majority of the prior studies analyzing the impact of CsA exposure have included a mix of hematological malignancies. As the GvL-effect and relapse tendency differs between diseases^50^, a comparison of the relapse risk between different diagnoses can be challenging. The refined Disease Risk Index have been used in some studies to compare relapse risk between diagnoses, but was actually developed to stratify patients to predict OS and not relapse per se^51^.

In our study, there is no clear reason why RFS and OS did not differ significantly between the studied groups, even though CIR was higher and relapse-related death more frequent, in the CsA_high_ group. An explanation may be that death from non-relapse causes was more common in the CsA_low_ group (42% versus 12%; p = 0.001), reflected by the numerically (n.s.) increased incidence of NRM in that group. Seven deaths from ”other” causes were seen in the CsA_low_ group, but none in the CsA_high_. It cannot be stated that the distribution of these deaths was purely coincidental, but no obvious explanatory association to CsA exposure can be made. These deaths have possibly contributed to NRM, resulting in similar RFS and OS. This is supported by the difference in TTR, in which relapse-free deaths were censored.

In contrast to many other studies, the differences in aGvHD were numerical, but not significant. The relatively small number of patients and the fact that our study was not designed for GvHD as primary endpoint, may both have contributed to this lack of difference. Furthermore, the length of CsA treatment was not registered, potentially affecting the results. It could also be speculated that ATG^25,29,52^ had a prophylactic effect against aGvHD, hypothetically reducing and evening out differences in aGvHD rates, otherwise seen without the impact of ATG.

Our study has limitations. Firstly, the retrospective design increases the risk of both known and unknown confounding factors. Secondly, the assessment of CsA median concentration to evaluate the CsA exposure, may be considered a weakness. For instance, utilizing the area under the curve may yield a more precise measure of CsA exposure. The concentration cut-off was chosen from EBMT recommendations and the total median CsA concentration in our study. However, the study findings remained consistent regardless of whether CsA exposure was defined using the concentration cut-off at 200 µg/L, quartiles or as a continuous variable. Additionally, post-hoc analysis of the non-linear effect of CsA concentration on relapse, revealed that the selected cut-off value was appropriate. Thirdly, we chose to study the impact of CsA exposure the first month after HSCT, but the treatment length and later CsA levels could potentially also have affected the reults. Finally, outcomes may have been affected by differences in methods and supportive care between the transplantation centres.

In conclusion, to our knowledge, this is one of few studies focusing on a uniform cohort exclusively consisting of AML patients, allografted with URD and T-cell depleted with ATG, analyzing the impact of early CsA exposure on the relapse incidence. EBMT guidelines recommends a CsA target concentration of 200–300 µg/L during the first month after HSCT. However, we found that a blood CsA concentration above 200 µg/L during the first month after HSCT results in a higher incidence of AML relapse compared to lower concentrations when combined with ATG treatment. Although this finding needs to be evaluated in trials with more numerous groups and other AML HSCT cohorts, it indicates that the currently recommended CsA concentration interval post-HSCT should be more differentiated.

### Supplementary Information

Supplementary Information.

## Data Availability

The dataset analysed during the current study is available from the corresponding author on reasonable request.

## References

[1] Webster JA, Luznik L, Gojo I (2021). Treatment of AML relapse after allo-HCT. Front. Oncol..

[2] Styczynski J (2016). Impact of donor Epstein–Barr virus serostatus on the incidence of graft-versus-host disease in patients with acute leukemia after hematopoietic stem-cell transplantation: A study from the acute leukemia and infectious diseases working parties of the European Society for Blood and Marrow Transplantation. J. Clin. Oncol..

[3] Horowitz M (2018). Epidemiology and biology of relapse after stem cell transplantation. Bone Marrow Transplant.

[4] Arai S (2015). Increasing incidence of chronic graft-versus-host disease in allogeneic transplantation: A report from the Center for International Blood and Marrow Transplant Research. Biol. Blood Marrow Transplant.

[5] Fraser CJ (2006). Impact of chronic graft-versus-host disease on the health status of hematopoietic cell transplantation survivors: A report from the Bone Marrow Transplant Survivor Study. Blood.

[6] Wingard JR (2011). Long-term survival and late deaths after allogeneic hematopoietic cell transplantation. J. Clin. Oncol..

[7] Martin PJ (2010). Life expectancy in patients surviving more than 5 years after hematopoietic cell transplantation. J. Clin. Oncol..

[8] Ayuk F (2015). Prognostic factors for survival of patients with newly diagnosed chronic GVHD according to NIH criteria. Ann. Hematol..

[9] Pidala J (2011). The global severity of chronic graft-versus-host disease, determined by National Institutes of Health consensus criteria, is associated with overall survival and non-relapse mortality. Haematologica.

[10] Malard F (2010). Impact of cyclosporine-A concentration on the incidence of severe acute graft-versus-host disease after allogeneic stem cell transplantation. Biol. Blood Marrow Transplant.

[11] Martin P (2003). Relationship between CsA trough blood concentration and severity of acute graft-versus-host disease after paediatric stem cell transplantation from matched-sibling or unrelated donors. Bone Marrow Transplant.

[12] Przepiorka D (1991). Cyclosporine and methylprednisolone after allogeneic marrow transplantation: association between low cyclosporine concentration and risk of acute graft-versus-host disease. Bone Marrow Transplant.

[13] Bianchi M (2019). Cyclosporine levels > 195 mug/L on day 10 post-transplant was associated with significantly reduced acute graft-versus-host disease following allogeneic hematopoietic stem cell transplantation. Ann. Hematol..

[14] Rogosheske JR (2014). Higher therapeutic CsA levels early post transplantation reduce risk of acute GVHD and improves survival. Bone Marrow Transplant.

[15] Garcia Cadenas I (2014). Impact of cyclosporine levels on the development of acute graft versus host disease after reduced intensity conditioning allogeneic stem cell transplantation. Mediators Inflamm..

[16] Heritier J (2022). Optimized cyclosporine starting dose may reduce risk of acute GvHD after allogeneic hematopoietic cell transplantation: A single-center cohort study. Bone Marrow Transplant.

[17] Kwan ACF (2022). Toward optimization of cyclosporine concentration target to prevent acute graft-versus-host disease following myeloablative allogeneic stem cell transplant. Clin. Transplant.

[18] Penack O (2020). Prophylaxis and management of graft versus host disease after stem-cell transplantation for haematological malignancies: Updated consensus recommendations of the European Society for Blood and Marrow Transplantation. Lancet Haematol..

[19] Bacigalupo A (1991). Increased risk of leukemia relapse with high-dose cyclosporine A after allogeneic marrow transplantation for acute leukemia. Blood.

[20] Locatelli F (2000). Graft versus host disease prophylaxis with low-dose cyclosporine-A reduces the risk of relapse in children with acute leukemia given HLA-identical sibling bone marrow transplantation: Results of a randomized trial. Blood.

[21] Bacigalupo A (2018). High vs low dose cyclosporine-A, after allogeneic marrow transplantation in leukemia: Long term follow up of a randomized study. Am. J. Hematol..

[22] Craddock C (2010). Factors predicting long-term survival after T-cell depleted reduced intensity allogeneic stem cell transplantation for acute myeloid leukemia. Haematologica.

[23] Bacigalupo A (2005). Antilymphocyte/thymocyte globulin for graft versus host disease prophylaxis: efficacy and side effects. Bone Marrow Transplant.

[24] Kroger N (2016). Antilymphocyte globulin for prevention of chronic graft-versus-host disease. N. Engl. J. Med..

[25] Walker I (2016). Pretreatment with anti-thymocyte globulin versus no anti-thymocyte globulin in patients with haematological malignancies undergoing haemopoietic cell transplantation from unrelated donors: A randomised, controlled, open-label, phase 3, multicentre trial. Lancet Oncol..

[26] Chang YJ (2020). Antithymocyte globulin for matched sibling donor transplantation in patients with hematologic malignancies: A multicenter, open-label, randomized controlled study. J. Clin. Oncol..

[27] Ruutu T (2012). Prophylaxis and treatment of GVHD after allogeneic haematopoietic SCT: A survey of centre strategies by the European Group for Blood and Marrow Transplantation. Bone Marrow Transplant.

[28] Kroger N (2016). Outcome improvement after allogeneic stem-cell transplantation in myelofibrosis. J. Oncol. Pract..

[29] Finke J (2009). Standard graft-versus-host disease prophylaxis with or without anti-T-cell globulin in haematopoietic cell transplantation from matched unrelated donors: A randomised, open-label, multicentre phase 3 trial. Lancet Oncol..

[30] Soiffer RJ (2017). Prospective, randomized, double-blind, phase III clinical trial of anti-T-lymphocyte globulin to assess impact on chronic graft-versus-host disease-free survival in patients undergoing HLA-matched unrelated myeloablative hematopoietic cell transplantation. J. Clin. Oncol..

[31] Devillier R (2018). Impact of antithymocyte globulin doses in reduced intensity conditioning before allogeneic transplantation from matched sibling donor for patients with acute myeloid leukemia: A report from the acute leukemia working party of European Group of Bone Marrow Transplantation. Bone Marrow Transplant.

[32] *Nationellt Vårdprogram för AML; Version 7.0*. (Regionala Cancercentrum i Samverkan, 2022).

[33] Schoemans HM (2018). EBMT-NIH-CIBMTR Task Force position statement on standardized terminology & guidance for graft-versus-host disease assessment. Bone Marrow Transplant.

[34] Jagasia MH (2015). National Institutes of Health consensus development project on criteria for clinical trials in chronic graft-versus-host disease: I. The 2014 diagnosis and staging working group report. Biol. Blood Marrow Transplant.

[35] Bacigalupo A (2009). Defining the intensity of conditioning regimens: Working definitions. Biol. Blood Marrow Transplant.

[36] Schemper M, Smith TL (1996). A note on quantifying follow-up in studies of failure time. Control Clin. Trials.

[37] Sahtish, N. W. C-L Ally. In *PharmaSUG 2019—Paper ST-81-5* (Philadelphia, 2019).

[38] Kim HT (2007). Cumulative incidence in competing risks data and competing risks regression analysis. Clin. Cancer Res..

[39] Gray RJ (1988). A class of K-sample tests for comparing the cumulative incidence of a competing risk. Ann. Stat..

[40] Fine JP, Gray RJ (1999). A proportional hazards model for the subdistribution of a competing risk. J. Am. Stat. Assoc..

[41] Lambert PCR (2009). Further development of flexible parametric models for survival analysis. Stata J..

[42] Passweg JR (2022). Impact of the SARS-CoV-2 pandemic on hematopoietic cell transplantation and cellular therapies in Europe 2020: A report from the EBMT activity survey. Bone Marrow Transplant.

[43] Bejanyan N (2015). Survival of patients with acute myeloid leukemia relapsing after allogeneic hematopoietic cell transplantation: A center for international blood and marrow transplant research study. Biol. Blood Marrow Transplant.

[44] Zuanelli Brambilla C (2021). Relapse after allogeneic stem cell transplantation of acute myelogenous leukemia and myelodysplastic syndrome and the importance of second cellular therapy. Transplant Cell Ther..

[45] Grimwade D (2010). Refinement of cytogenetic classification in acute myeloid leukemia: Determination of prognostic significance of rare recurring chromosomal abnormalities among 5876 younger adult patients treated in the United Kingdom Medical Research Council trials. Blood.

[46] Grimwade D, Ivey A, Huntly BJ (2016). Molecular landscape of acute myeloid leukemia in younger adults and its clinical relevance. Blood.

[47] Tallman MS (2007). Impact of cytogenetics on outcome of matched unrelated donor hematopoietic stem cell transplantation for acute myeloid leukemia in first or second complete remission. Blood.

[48] Dohner H, Weisdorf DJ, Bloomfield CD (2015). Acute myeloid leukemia. N. Engl. J. Med..

[49] Wong R (2005). Prognostic factors for outcomes of patients with refractory or relapsed acute myelogenous leukemia or myelodysplastic syndromes undergoing allogeneic progenitor cell transplantation. Biol. Blood Marrow Transplant.

[50] Kolb HJ (2008). Graft-versus-leukemia effects of transplantation and donor lymphocytes. Blood.

[51] Armand P (2014). Validation and refinement of the Disease Risk Index for allogeneic stem cell transplantation. Blood.

[52] Theurich S (2012). Polyclonal anti-thymocyte globulins for the prophylaxis of graft-versus-host disease after allogeneic stem cell or bone marrow transplantation in adults. Cochrane Database Syst. Rev..

[53] Bjork, J. *et al.* Revised equations for estimating glomerular filtration rate based on the Lund-Malmö Study cohort. *Scand J Clin Lab Invest* **71**, 232–239. 10.3109/00365513.2011.557086 (2011). 10.3109/00365513.2011.557086 21391777

