# Supplementary material for: Higher cyclosporine-A concentration increases the risk of relapse in AML following allogeneic stem cell transplantation from unrelated donors using anti-thymocyte globulin
Source: Sci Rep. 2023 Dec 20;13:22777. doi: 10.1038/s41598-023-50105-4 (PMC10733303; doi:10.1038/s41598-023-50105-4)
Supplement: Supplementary file 1 — Supplementary Information. [file 41598_2023_50105_MOESM1_ESM.pdf]

Supplementary 1. Methods for analyzing CsA concentration

Electrochemiluminescence immunoassay (ECLIA) or chemiluminescent microparticle immunoassay (CMIA) was used to analyze CsA concentration. In one centre (Lund) liquid chromatography tande masspectrometry (LC-MS) was also used for lower values.

Methods and changes during the studied period:

Gothenburg
04 June 2015: Change from Architect® on instrument Abbott® TDx (CMIA) to
Elecsys Cyclosporine® on instrument Cobas® e602 (Roche Diagnostics) (ECLIA)

Lund
February 2014:
Change from Cedia® Cyclosporine Plus on instrument Cobas® c502 (Roche Diagnostics) to Elecsys Cyclosporine® on instrument Cobas® e601/602 (Roche Diagnostics) (ECLIA)

Parallell method (low values):
HPLC Nexera (Shimadzu) and QTrap 5500 (AB Sciex) on a Kinetex Biphenyl column (Phenomenex) (LC-MS/MS)

Umeå:
14 October 2010 Change from Architect® on instrument Abbot® TDx to
Cedia® Microgenics on instrument Cobas® c6000 (Roche Diagnostics)
17 November 2015 Change to Elecsys® Cyclosporine on instrument Cobas® e602 (Roche Diagnostics) (ECLIA)

Supplementary 2. Risk Categorization

AML risk categorization according to the Swedish national AML guidelines

Genetic low risk (LR)

- APL with t(15;17)(q24;q21), or molecularly detected PML/RARA-fusion. In rare cases there are variant translocations of RARA present. Theese are categorized as low risk, also in the presence of other concomitant cromosomal abnormalities.
- inv16(p13q22)/t(16;16)(p13;q22), or molecularly detected CBFB/MYH11–fusion or CBFB-rearrangement. Theese are classified as low risk, also in the presence of other concomitant cromosomal abnormalities.
- Exception: Not categorized as low risk if KIT-mutation is detected.
- t(8;21)(q22;q22), alternatively molecularly detected RUNX1/RUNX1T1-fusion (formerly AML/ETO). Theese are categorized as low risk, also in the presence of other concomitant cromosomal abnormalities.
- *Mutated NPM1 concomitant wild type FLT3-ITD and normal karyotype.*
- Double mutation of CEBPA and normal karyotype.

Genetic intermediate risk (IR)

- Normal karyotype without any concomitant abnormalities: FLT3-ITD, mutated NPM1 or double mutation of CEBPA.
- *Normal karyotype with concomitant mutation of both NPM1 and FLT3-ITD.*
- t(9;11)(p21;q23).
- Cytogenetic abnormality (karyotype) neither categorized as low or high risk.

Genetic high risk (HR)

- *Normal or not-riskcategorizing karyotype with concomitant mutation of FLT3-ITD and wild type NPM1.*
- inv(3)(q21q26) or t(3;3)(q21;q26); GATA2::MECOM (formerly EVI1).
- t(6;9)(p22;q34); DEK/NUP214.
- t(v;11)(v;q23); KMT2A-rearrangement (formerly classified MLL-rearrangement). Exception: t(9;11)(p21;q23) is categorized as intermediate risk.
- Isolated del(5q) or -5 or with other concomitant abnormalities.
- Isolated del(7q) or -7 or with other concomitant abnormalities.
- del(17p).
- Complex karyotype, defined as ≥3 cromosomal abnormalities in the absence of t(15;17)(q22;q21), t(8;21)(q22;q22), inv(16)(p13q22)/t(16;16)(p13;q22), or t(9;11)(p21;q23).
- TP53 mutation (usually associated with complex and/or monosomal caryotype)

Special cases: Normal Karyotype (NK), or Not Done (ND), and different findings of NPM1 & FLT3

Cg NK or ND	FLT3	NPM1	Risk group
Cg NK/ND	pos	ND	IR
Cg NK/ND	pos	neg	HR
Cg NK/ND	pos	pos	IR
Cg NK/ND	neg	ND	IR
Cg NK/ND	neg	neg	IR
Cg NK	neg	pos	LR
Cg ND	neg	pos	IR
Cg NK/ND	ND	ND	IR
Cg NK/ND	ND	neg	IR
Cg NK/ND	ND	pos	IR

- FLT3- and NPM1-status are only informative, considering risk categorization, if both are concomitantly analyzed.
- Cytogenetics ND is always categorized as intermediate risk unless mutated FLT3.

Comment: Final risk categorization (in the CRF) also includes clinical assessment. Secondary AML (secondary to other hematological disease), therapy related AML, refractory AML (>15% blasts in bone marrow after first cycle of chemotherapy or >2 cycles of chemotherapy needed to achieve complete remission) and all other disease status than CR1 (incl CR2), are always categorized as high risk.

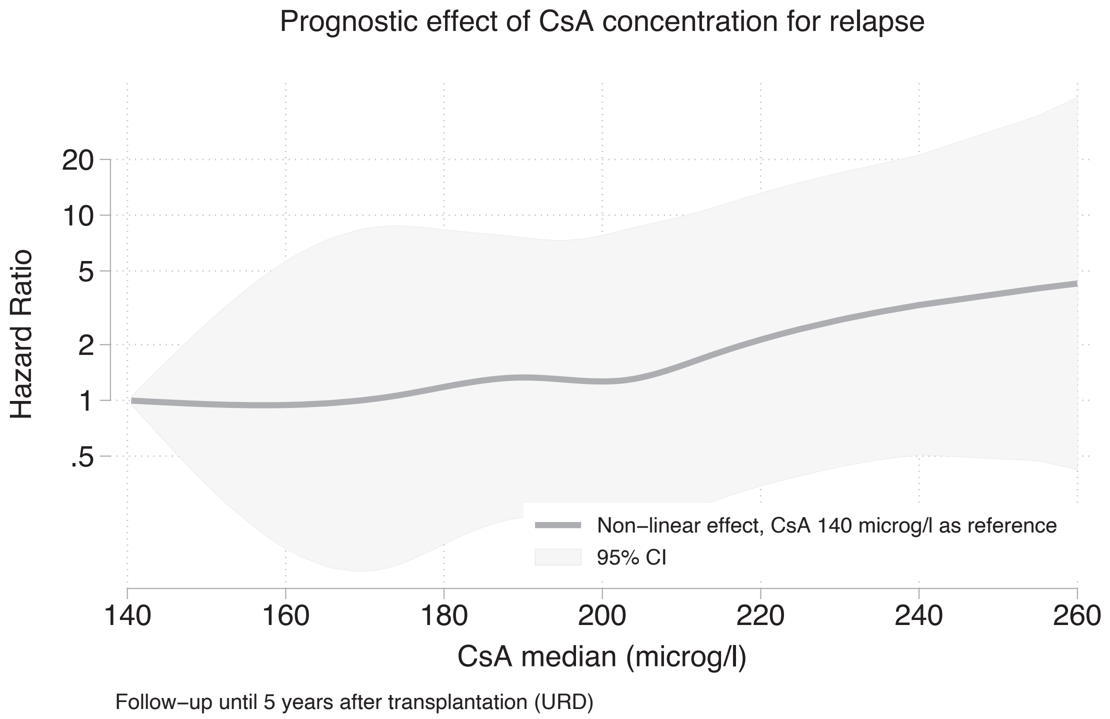

Supplementary Figure 1 Prognostic non-linear effect of CsA on relapse during the first 60 months post-HSCT.

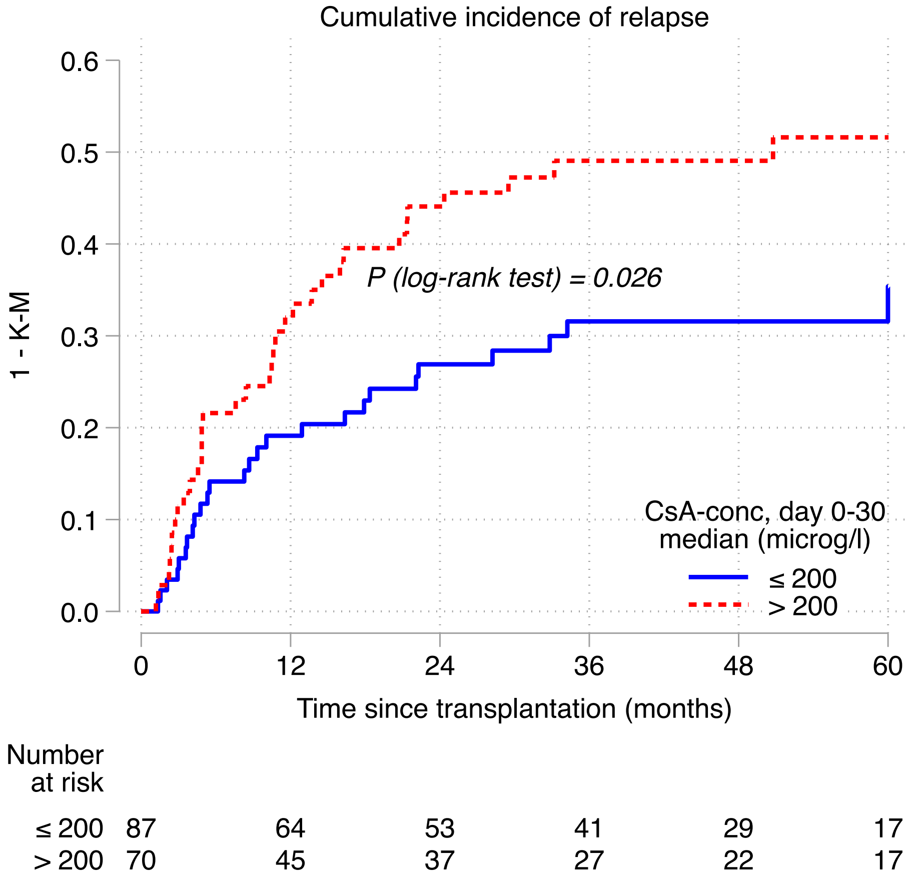

Supplementary Figure 2 The cumulative incidence of relapse during the first 60 months post-HSCT, compared between CsA$_{high}$ and CsA$_{low}$. Relapse-free deaths censored.
